# Art therapy, occupational and play therapy used as multidisciplinary tools for prevention and early recovery for children and adolescents at risk of developing mental health problems and juvenile deliverance

**DOI:** 10.1192/j.eurpsy.2023.1733

**Published:** 2023-07-19

**Authors:** C. Emilia

**Affiliations:** Centru Comunitar Județean Complex Servicii Sociale Comunitare pentru Copii și Adulți Cluj, CONSILIUL JUDEȚEAN CLUJ DIRECŢIA GENERALĂ DE ASISTENŢĂ SOCIALĂ ŞI PROTECŢIA COPILULUI CLUJ, Cluj-Napoca, Romania

## Abstract

**Introduction:**

The new type of postmodern art can be interpreted from the point of view of self expression polarized between: art - art-therapy, ludic, occupational therapy - religion. The computer becomes the new medium after the impact of television, and by integrating computers into the network, their communication function has become more prevalent than that of data processing. Contemporary visual arts bring together, in different degrees of relationship and fusion, fields of art that until now were understood and practiced more individually. Multimedia and mixed media technology, which has evolved into meaningful visual representations, incorporates the science behind human perception and knowledge.

**Objectives:**

The purpose of art in art-therapy, in this context, is not an exercise of the already acquired knowledge upon the artistic material, but a discovery of the yet unknown. Art-based therapies, as nondirective methods, attempt to visualize past traumatic experiences and harmonize the individual with himself and with others.

**Methods:**

In the preventive activities, we include all activities involving nonverbal communication and holistic engagement of people in creative activities, specific to visual arts (plastic, decorative design, and multimedia).”*Beneficiaries can create their own images with which they want to interact, to arrange their environment…We experiment with art-specific ways to make interdisciplinary exchanges and cultural interferences using the universal language of visual arts along with intercultural elements and religious ecumenism”* . The child expresses various issues related to his feelings, like the search of his identity, the generated anxieties, the family and professional environment, the situations of neglect and abuse. For the same purpose, as a complement to activities in nature camps, where education/art therapy takes place in the artist’s studio, special care must be taken to create an evocative and stimulating work environment.

**Results:**

We detect hidden capacities through the specific means of the visual arts with the aim of providing the freedom to follow one’s own destiny, encouraging the joy of creatively manifesting at multiple levels of difficulty in any activity. Harmonizing cultural differences develop self-esteem, tolerance, resilience, and necessary adaptation to the conditions of a multiethnic society.

**Image:**

**Figure:**
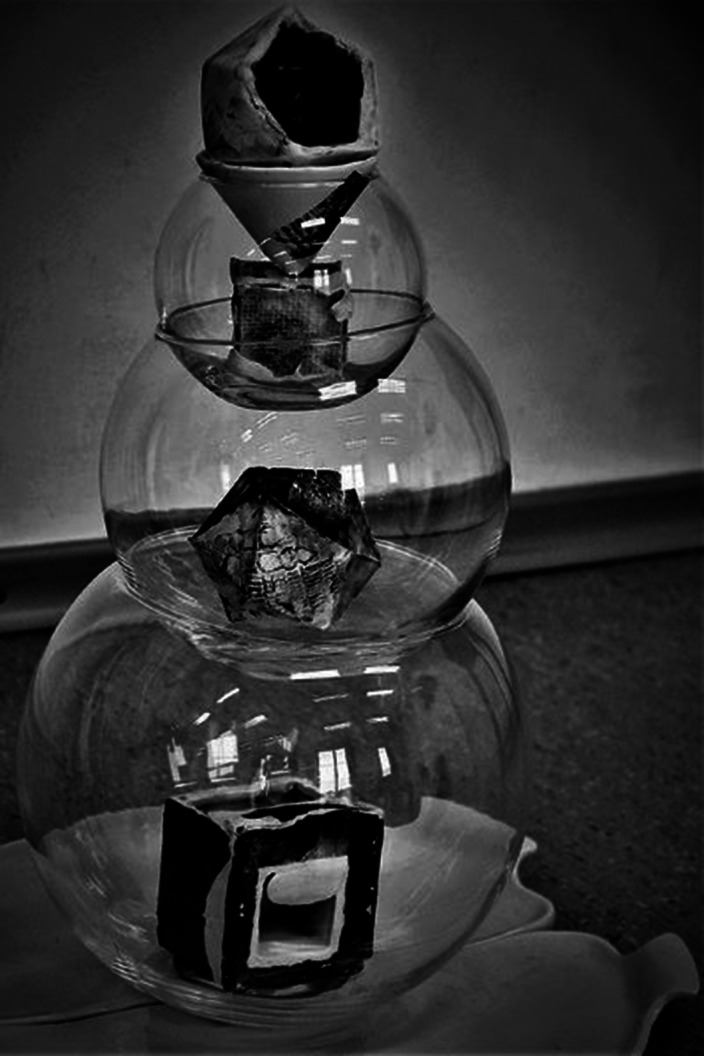

**Conclusions:**

There is an equidistant trialogue and circular relations between art, religion, and science, without any specific supremacy. This can offer up from the start the possibility of a lasting harmonization, of information transfers and professional enhancements to support new developments, uplifting the human being through positive reorientations and beneficial recoveries.

**Disclosure of Interest:**

None Declared

